# EARLY DETECTION OF DEMENTIA AND MILD COGNITIVE IMPAIRMENT WITH BRAINCHECK

**DOI:** 10.1093/geroni/igad104.2406

**Published:** 2023-12-21

**Authors:** Bin Huang, Duong Huynh, Reza Hosseini Ghomi

**Affiliations:** BrainCheck, Austin, Texas, United States; BrainCheck, Austin, Texas, United States; BrainCheck, Austin, Texas, United States

## Abstract

Dementia is projected to affect 14 million people in the United States and 152 million people globally in the coming decades. Early detection of dementia, which involves diagnosis of mild cognitive impairment (MCI), a possible prestage of dementia, is critical for intervention and care planning but remains a challenge with current clinical protocols. While brief screening tools are narrow in scope, comprehensive neuropsychological tests are time-consuming and require specialized training and expertise to conduct and interpret results. BrainCheck, a computerized cognitive assessment tool, provides an accessible and promising solution to address these current challenges. The aim of this study was to evaluate BrainCheck for its diagnostic accuracy and ability to distinguish the severity of cognitive impairment. A total of 99 participants clinically diagnosed with dementia (DEM; n=42), MCI (n=22), or normal cognition (NC; n=35) completed the BrainCheck battery. Statistical analyses compared participant performances on BrainCheck based on their diagnostic group. BrainCheck battery performance showed significant differences between the three groups, achieving 88% or higher sensitivity and specificity for separating DEM from NC, and 77% or higher sensitivity and specificity in separating the MCI group from the NC and DEM groups. Three-group classification found true positive rates of 80% or higher for the NC and dementia groups and true positive rates of 64% or higher for the MCI group. These results show that BrainCheck was able to distinguish between diagnoses of DEM, MCI, and NC, providing a potentially reliable tool for early detection of cognitive impairment.

